# Genome-Wide Identification and Analysis of the EPF Gene Family in *Sorghum bicolor* (L.) Moench

**DOI:** 10.3390/plants12223912

**Published:** 2023-11-20

**Authors:** Zhiyin Jiao, Jinping Wang, Yannan Shi, Zhifang Wang, Jing Zhang, Qi Du, Bocheng Liu, Xinyue Jia, Jingtian Niu, Chun Gu, Peng Lv

**Affiliations:** 1 Institute of Millet Crops, Hebei Academy of Agriculture and Forestry Sciences/Hebei Branch of National Sorghum Improvement Center/Key Laboratory of Genetic Improvement and Utilization for Featured Coarse Cereals (Co-Construction by Ministry and Province), Ministry of Agriculture and Rural Affairs/Key Laboratory of Minor Cereal Crops of Hebei Province, Shijiazhuang 050035, China; 2 Hebei Seed Management Station, Shijiazhuang 050031, China; hbzyfz@163.com; 3 Hebei Xingtang County Agro-Technology Extension Center, Shijiazhuang 050600, China

**Keywords:** EPIDERMAL PATTERNING FACTOR (EPF), *Sorghum bicolor* (L.) Moench, bioinformatics, drought stress

## Abstract

The EPIDERMAL PATTERNING FACTOR (EPF) plays a crucial role in plant response to abiotic stress. While the EPF has been extensively studied in model plants such as *Arabidopsis thaliana*, there is a lack of research on identifying EPF genes in the whole sorghum genome and its response to drought stress. In this study, we employed bioinformatics tools to identify 12 EPF members in sorghum. Phylogenetic tree analysis revealed that SbEPFs can be categorized into four branches. Further examination of the gene structure and protein conservation motifs of EPF family members demonstrated the high conservation of the SbEPF sequence. The promoter region of SbEPFs was found to encompass cis-elements responsive to stress and plant hormones. Moreover, real-time fluorescence quantitative results indicated that the SbEPFs have a tissue-specific expression. Under drought stress treatment, most SbEPF members were significantly up-regulated, indicating their potential role in drought response. Our research findings establish a foundation for investigating the function of SbEPFs and offer candidate genes for stress-resistant breeding and enhanced production in sorghum.

## 1. Introduction

Plants inhabit a dynamic environment, necessitating their response to diverse biotic and abiotic stresses [1,2]. Notably, drought stands as the foremost environmental stressor impacting both plant growth and crop production [1,3]. It represents a paramount factor limiting global crop yield, rendering it a pressing challenge for agricultural production worldwide [3]. Throughout their growth cycle, crops demand ample water and nutrients. Diminished soil moisture levels can induce profound physiological and biochemical alterations in crop physiology, ultimately impacting yield and quality [4]. Over the last decade, the severity of global crop losses attributed to drought has been significant [5]. Nevertheless, the anticipated repercussions of climate change indicate a projected 50% reduction in freshwater availability by 2050 [6]. Consequently, the enhancement of crop drought resistance has emerged as a pressing scientific quandary necessitating immediate attention.

Sorghum [*Sorghum bicolor* (L.) Moench], an important C_4_ cereal, serves as the primary food crop cultivated in arid and semi-arid regions. The yields of these crops face escalating risks due to the mounting severity of water scarcity in these regions [4,7]. Particularly, sorghum stands out as an exceptionally drought-tolerant crop, holding a prominent role in our traditional rainfed agriculture. Moreover, it is increasingly recognized as a paradigmatic grain for imparting drought resistance [7]. However, different sorghum varieties have varying degrees of drought resistance. Exploring the drought resistance mechanism of sorghum and studying its key drought resistance genes is not only beneficial for the innovation of sorghum drought resistance germplasm, but also provides a theoretical basis for its application to other crops.

The EPIDERMAL PATTERNING FACTOR (EPF) family comprises a collection of cysteine-rich secreted peptides that wield a pivotal role in stomatal development, thereby contributing to plant growth, development, and stress response [8]. EPF genes are found across mosses, monocots, and dicots, indicating their widespread occurrence in the plant kingdom [9]. The EPF family encompasses 11 members in *Arabidopsis*, including *EPF1*, *EPF2*, and *EPFL1-9*. *AtEPF1*, *AtEPF2*, *AtEPFL6*, and *AtEPFL9* have been confirmed to play a vital role in stomatal development [10,11]. The regulation of stomatal development, encompassing stomatal density and size, presents an efficacious strategy for mitigating drought stress by curtailing transpiration water loss and augmenting plant water use efficiency (WUE) [12,13,14].

Several studies have demonstrated the involvement of EPF family members in the response to various plant stresses. For instance, *EPF1* has been found to reduce stomatal density in rice, barley, and wheat, thereby enhancing drought tolerance [15,16,17]. Overexpression of *StEPF2* in *Arabidopsis* significantly decreases stomatal density and imparts higher photosynthetic rates, photosystem II efficiency, and instantaneous water use efficiency compared to wild-type plants under drought stress [18]. Recent studies have revealed the significant roles played by *EPF* gene family members in the growth and development of poaceae plants. In rice, the overexpression of the homologous gene *EPFL1* induces awn elongation, while the specific splicing of the RAE2 precursor by SLP1 in rice spikelets triggers the elongation of mature RAE2 peptides [19]. Additionally, another member of the EPFL family, GAD1/RAE2, encodes a secreted peptide that regulates rice grain number, grain length, and awn development [20]. Kawamoto et al. [21] discovered that EPFL2 and EPFL9 collaborate in coordinating embryo sac spacing and fruit seed number and growth by interacting with the ERECTA family receptor. *EPFL2* primarily governs the formation and spacing of embryo sac primordia during pistil and fruit growth through *ERL1* and *ERL2*, while *EPFL9* facilitates fruit growth via the endoplasmic reticulum. By employing CRISPR/Cas9 gene editing technology in the FAZ1 background of japonica rice, the knockout of small peptide encoding genes such as *OsEPFL5*, *OsEPFL6*, *OsEPFL7*, *OsEPFL8*, and *OsEPFL9* had varying degrees of impact on yield-related traits, such as grain number per panicle, grain size, and fertility. Specific inhibition of the OsEPFL6-OsER1, OsEPFL7-OsER1, and OsEPFL9-OsER1 ligand-receptor pairs can optimize rice panicle structure and achieve the desired panicle type with high yield [22]. These research findings indicated that EPF family members serve as promising candidate genes, demonstrating potential for both high yield and drought resistance.

With the completion of sorghum genome sequencing, an increasing number of functional genes have been identified. However, there is currently a lack of research on the EPF in sorghum. In this study, the identification of the sorghum EPF gene family, including its chromosomal location, gene structure, conserved domains, cis-regulatory elements in the promoter, and evolutionary relationships of homologous genes, were analyzed. Additionally, the study investigated the tissue-specific expression pattern and transcriptional response to drought stress of this gene family, providing a theoretical foundation for elucidating the function of *SbEPFs* in sorghum and informing future sorghum breeding efforts.

## 2. Results

### 2.1. Identification and Physicochemical Analysis of the Sorghum EPF

**Figure 1:**
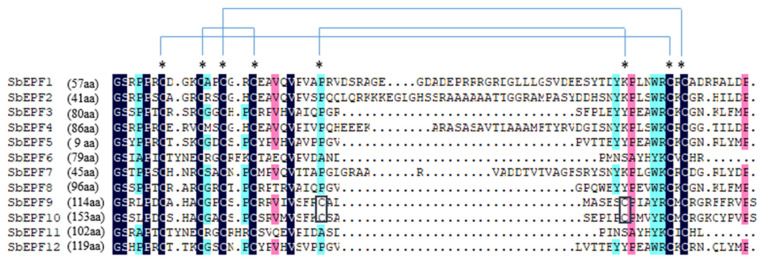
Multiple alignment of the C-terminal region between SbEPF protein sequences. Parentheses, number of omitted amino acid residues; *, conserved cysteine residues; blue line, predicted pairs of cysteine residue forming disulfide bonds.

The gene family was identified by searching with information probes that included 11 members of the EPF family in *Arabidopsis* and the conserved domain of the EPF family (pfam17181), resulting in the identification of 12 EPF members in sorghum. Detailed information on the gene ID, amino acid length, molecular weight, isoelectric point (PI), and other characteristics of each *SbEPF* family member is provided in Table 1. As shown in Table 1, all SbEPF family proteins are relatively short, with lengths ranging from 111aa (Sobic.004G229700) to 169aa (Sobic.009G173200), with corresponding molecular weights ranging from 11.784 to 18.181 KDa, and isoelectric points ranging from 7.61 to 11.51. Generally, studying the molecular characteristics of SbEPFs contributes to the understanding of their specific biological functions. The results of multiple sequence alignment demonstrate that SbEPF family members possess 6–8 conserved cysteine residues at the C-terminus (Figure 1).

### 2.2. The Chromosomal Localization of the SbEPF Gene Family

**Figure 2:**
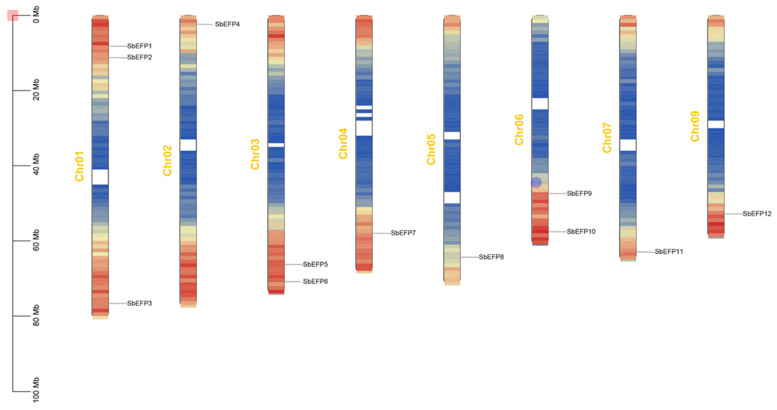
Chromosomal localization of SbEPF family in Sorghum. Size of each chromosome and gene position can be estimated according to the scale on the left of the figure. Chromosome colors represent gene abundance.

The nomenclature of the *SbEPF* gene family was based on their physical positions on the chromosomes, starting from the upper arm of chromosome 1 and moving downwards to the lower arm (*SbEPF1* to *SbEPF12*) (Figure 2). The analysis of chromosomal localization showed that the 12 *SbEPFs* were distributed across eight chromosomes of sorghum, as depicted in Figure 2. The distribution of these genes on the chromosomes was uneven, ranging from one to three *SbEPF* genes. Chromosome 1 had the highest distribution, with three genes, followed by chromosomes 3 and 6, which had two genes each. Chromosomes 2, 4, 5, 7, and 9 contained one *SbEPF* gene each, while no *SbEPF* genes were found on chromosomes 8 and 10. There were no observed gene clustering events on the chromosomes.

### 2.3. Evolutionary Analysis of Arabidopsis and Sorghum EPF

**Figure 3:**
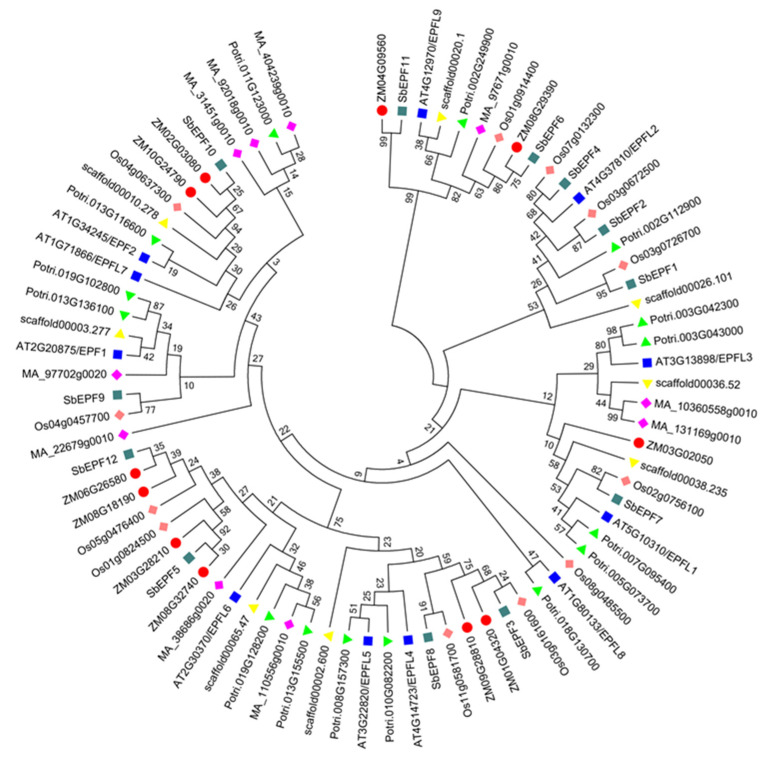
Phylogenetic analysis of EPF family genes in *P. trichocarpa*, *Zea mays*, *Picea abies*, *Oryza sativa*, *Amborella trichopoda*, and *Arabidopsis thaliana*. Different species are indicated by different colors.

To investigate the evolutionary and divergent relationships of EPF family proteins across species, the amino acid sequences of 11 *Arabidopsis* EPF protein sequences were used as probes, and EPF family members from other species were identified through BLAST comparisons. There are 12 SbEPF family members in Sorghum, and 15, 11, 11, 12, and 6 EPF family members in *poplar*, *Zea mays*, *Picea abies*, *Oryza sativa*, and *Amborella trichopoda*, respectively. Using MEGA6 and the NJ method, multiple sequence alignment of full-length EPF proteins from these seven species was performed, and a phylogenetic tree was constructed. According to the classification in *Arabidopsis* [9], the SbEPFs were divided into four sub-branches: I, II, III, and IV (Figure 3), consisting of *SbEPF9* and *SbEPF10* (homologous to *AtEPF1*-*EPF2*-*EPFL7*); *SbEPF1*, *SbEPF2*, *SbEPF4*, and *SbEPF7* (*AtEPFL1*-*EPFL3-EPFL8*); *SbEPF3, SbEPF5*, *SbEPF8*, and *SbEPF12* (*AtEPFL4*-*EPFL6*); and *SbEPF6* and *SbEPF11* (*AtEPFL9*), respectively. Overall, the classification of SbEPFs confirmed their diversification, indicating that different family members may possess distinct functions.

### 2.4. The Gene Structure and Protein Conserved Motifs Analysis of the Sorghum EPF Family Members

**Figure 4:**
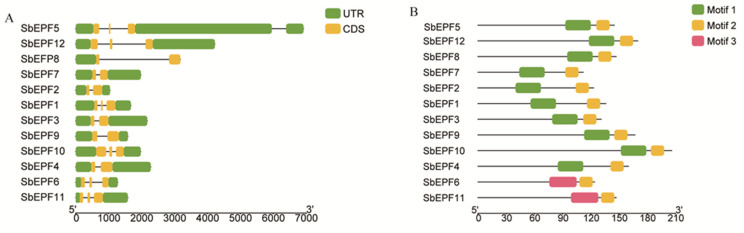
Gene structure and conserved protein motifs analysis of members in SbEPF family. (**A**) Gene structures of *SbEPFs*. (**B**) Conserved motifs of SbEPFs.

In order to investigate the evolution and specific features of the 12 sorghum EPF family members, we conducted an analysis of the gene structure and protein conserved motifs of the SbEPF family members. The results of the GSDS analysis showed that the length of the 5′-UTR or 3′-UTR varied among different *SbEPFs*, Additionally, each gene comprised 2–3 exons separated by introns of varying lengths (Figure 4A). The MEME analysis of the SbEPF protein sequences revealed that the conserved motifs of the SbEPFs were concentrated in the C-terminus, and most SbEPF members had a similar motif composition (Figure 4B). Except for *SbEPF6* and *SbEPF11*, all other EPF family members exhibited the presence of motif 1 and motif 2 (Figure 4B). The results of the GSDS and MEME analyses showed that *SbEPF6* and *SbEPF11*, which correspond to the only positive regulator of stomatal development in *Arabidopsis*, *AtEPFL9,* had the lowest conservation compared to other EPF family members, while other EPF family members had higher conservation. This discrepancy in conservation levels may explain the divergence in functions observed among EPF family members.

### 2.5. The EPF Genes’ Relationship between Sorghum and Arabidopsis

**Figure 5:**
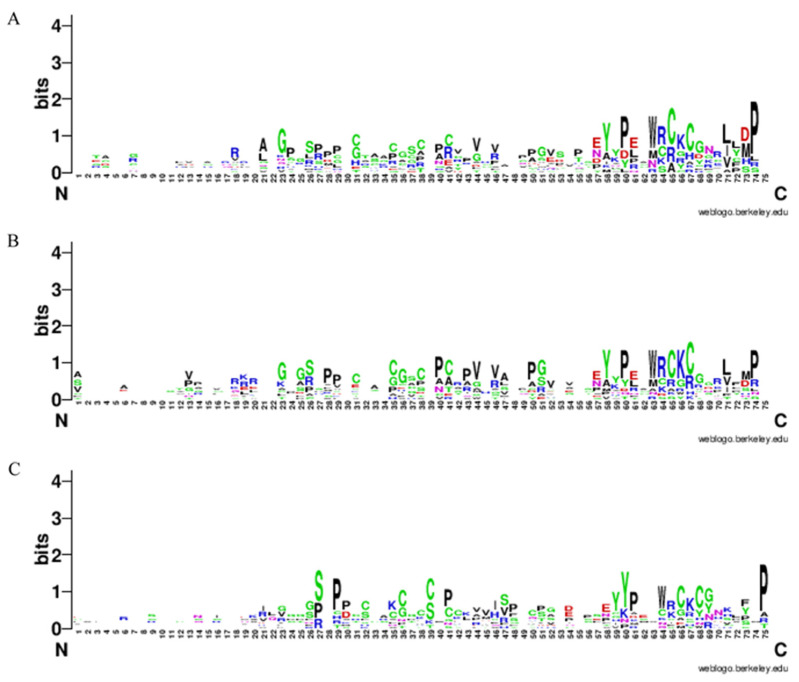
Logo analysis of EPF family conserved domain in (**A**) Sorghum (**B**) Rice and (**C**) Arabidopsis. The overall height of each stack indicates the sequence conservation at that point. The height of each letter (representing a residue) shows the relative frequency of the corresponding residue at that position.

The Sequence WebLogo Chart is commonly used to analyze and display the conservation of sequence patterns, with the height of each letter representing the relative frequency of the corresponding amino acid residue. Based on the conserved protein sequences of each member of the sorghum, rice, and Arabidopsis EPF family, as determined by NCBI’s CD-search, a uniform selection of the C-terminal 75 amino acids length was used for comparison. The results of the WebLogo Chart analysis depicted the composition, type, and site similarity of the conserved amino acid sequences among the 12 sorghum, 12 rice, and 11 *Arabidopsis* EPF members (Figure 5). The results showed that the composition of the conserved sequence of EPF protein, the types of conserved amino acids, and the proportion of site amino acids in sorghum, rice and *Arabidopsis* were very similar. Among the three, the similarity of EPF amino acids in sorghum and rice was higher.

### 2.6. Analysis of the SbEPF Gene Family Promoter

**Figure 6:**
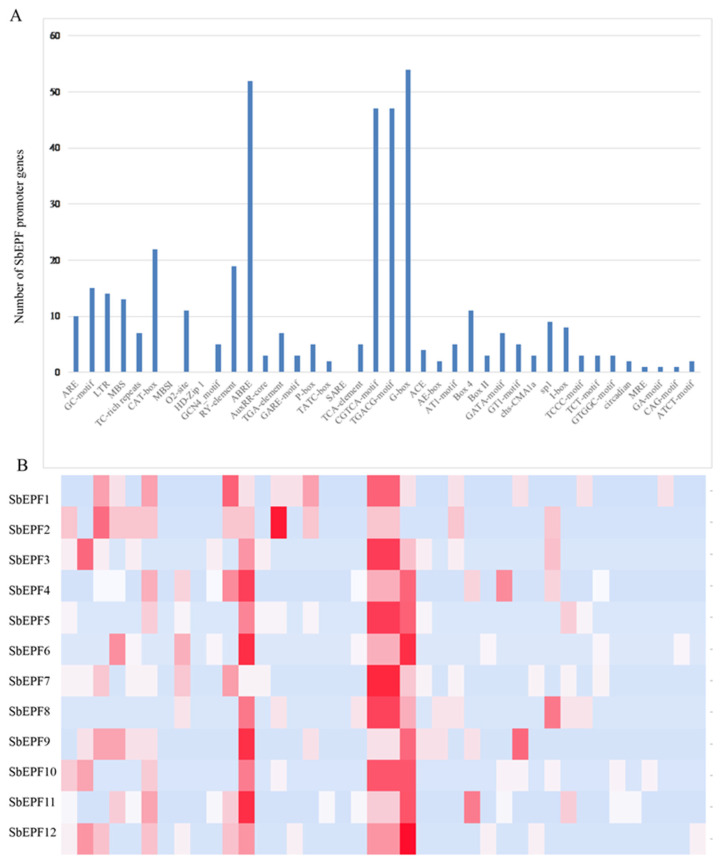
Analysis of cis-acting elements of promoter of EPF family gene in Sorghum. (**A**) Number of *SbEPF* prompter containing cis-acting elements. (**B**) The occurrences frequency of each cis-acting element in the promoter region of each category. The colors in the grid represent the number of cis-acting element.

To investigate the potential biological functions of the members of the SbEPF gene family, we conducted further prediction and analysis of their promoters. As shown in Figure 6, the cis-acting elements within the *SbEPF* gene family promoters can be broadly categorized into four groups: stress response elements, growth and development-related elements, plant hormone response elements, and light cycle-related elements. Stress response elements associated with abiotic stress include the drought response element (MBS), the anaerobic induction element (ARE), the low temperature response element (LTR), the defense stress response element (TC-rich), and the GC-motif, were found in about half of *SbEPF* promoters. Growth and development-related elements comprise the CAT-box associated with meristematic tissue expression, the GCN4_motif related to endosperm, the RY-element seed-specific regulatory element, and the regulation of corn alcohol-soluble protein metabolism (O_2_-site). Hormone response elements consist of the AuxRR-core and TGA-element associated with auxin, the ABA response element (ABRE), the salicylic acid response elements (SARE and TCA-element), the GARE-motif and P-box associated with gibberellin, and the CGTCA-motif and TGACG-motif associated with jasmonic acid (MeJA) response elements. All members have the ABRE- and MeJA-responsive elements, indicating that most SbEPFs were involved in plant hormones signal transduction. Only *SbEPF11* has the TCA-element. Additionally, there are several elements involved in light response. The total number and types of cis-acting elements in the EPF gene family promoter vary among different genes, suggesting that the diversity of these elements may contribute to the response of EPF gene family members to plants.

### 2.7. The Expression Profile of SbEPFs in Response to Various Abiotic Stresses

**Figure 7:**
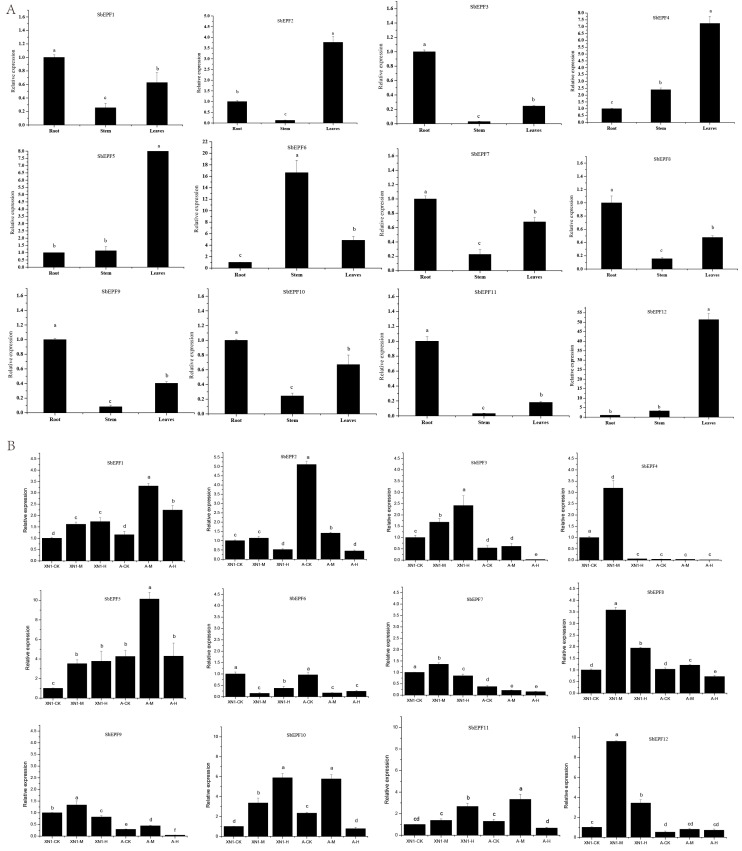
Tissue-specific expression profiles of *SbEPF* family. (**A**) Expression level of *SbEPFs* in different sorghum tissues (**B**) Expression level of *SbEPFs* under drought stress in sorghum (drought stress line: XN1; drought susceptible line: Aihongmao). Different lowercase letters indicated significant differences at 0.05 level.

To study potential biological functions of *SbEPFs*, the qRT-PCR was applied to determine the relative transcription level in various tissues of *SbEPF* in sorghum. The result revealed that the expression of *SbEPFs* has significant tissue specificity (Figure 7A). Most of the *SbEPF* genes were expressed in all organs. Among them, *SbEPF6* was mainly expressed in the stem tissue, and *SbEPF2*, *SbEPF4*, *SbEPF5* and *SbEPF12* were expressed in the leaves. Others *SbEPFs* had the highest expression level in the roots. Furthermore, in order to estimate the response mechanism of *SbEPFs* to drought stress treatment, two varieties of sorghum with different resistance were subjected to drought stress treatment. It can be seen that after drought treatment, some *SbEPF* genes in sorghum were up-regulated and some were down-regulated, and the space and time expression of each member was different (Figure 7B). For example, the transcription abundance of *SbEPF1*, *SbEPF5*, *SbEPF8*, *SbEPF9*, *SbEPF10*, and *SbEPF11* were significantly induced by drought treatment in both the drought-tolerant sorghum variety and the drought-sensitive variety. A significant up-regulation was observed for *SbEPF8* and *SbEPF9* under moderate drought in both cultivars, while their expression decreased after heavy drought stress. The expression patterns of *SbEPF1*, *SbEPF5*, and *SbEPF10* are similar, with a trend of up-regulation followed by down-regulation in drought sensitive varieties. In drought tolerant sorghum varieties, these genes were up-regulated when subjected to moderate drought. *SbEPF12* transcription abundance peaked under moderate drought stress, which was 9.6 fold higher than the control in drought-tolerant sorghum. However, *SbEPF6* was significantly decreased in the drought response of the two varieties. In summary, the expression patterns of these *SbEPFs* under drought stress conditions indicated that different *SbEPFs* may play an important role in drought stress tolerance.

## 3. Discussion

EPFs, which are small secreted peptides widely distributed in plants, play a crucial role in plant growth, development, and stress tolerance. Advances in sequencing technologies have led to the identification of an increasing number of EPF genes in various plants, such as poplar, Arabidopsis, tomato, and rice [20,23,24]. Sorghum, as a typical C_4_ crop, holds significant importance as a raw material in animal husbandry, nutrition, and brewing industries. Despite the publication of the reference genome of sorghum [25], a comprehensive understanding of the EPF gene family in sorghum is still lacking.

In this study, 12 EPF genes were identified in sorghum plants. According to previous studies on gene families [26], *SbEPFs* were named *SbEPF1*-*SbEPF12* based on their chromosomal location (Table 1). SbEPF family members exhibit relatively short protein lengths and similar molecular weights (Table 1 and Table S1). Analysis of the gene structure and protein motifs revealed that most SbEPFs within each subfamily have 1–3 introns, and the number and distribution of exons and introns are relatively consistent (Figure 4). EPF family genes are highly conserved only in their C-terminal region (Figure 1). Furthermore, the distribution of conserved protein motifs among EPF family members aligns with the branch divisions in the phylogenetic tree. Based on the phylogenetic tree (Figure 3 and Figure 4B), these results support the classification of 12 SbEPF members into four subfamilies (I, II, III, and IV). The EPF family is conserved in land plants but not in algae, and there are no members of the II and IV branches in moss, suggesting that these two branches may have originated after ferns [27]. The results from the phylogenetic tree indicate an increase in the number of EPF family members during plant evolution, potentially driven by gene duplication, which could serve as the primary source of plant evolution and genetic variation (Figure 2). EPFs possess six conserved cysteine residues at their C-terminus. Among SbEPFs, SbEPF9 and SbEPF10 (EPF1-EPF2-EPFL7 sub-branch) exclusively contain two additional conserved cysteine residues. These cysteine residues have the ability to form three or four pairs of disulfide bonds, which play a crucial role in the structure and functional activity of EPF proteins and are essential for the formation of a consensus scaffold. The absence of disulfide bonds can lead to protein misfolding and inactivation, which is likely the site where the family protein interacts with downstream protein kinase receptors [28,29,30,31]. Among the sub-branches in sorghum, both sub-branches I and III consist of two members each, collectively representing 16.67% of all SbEPFs. The II and IV sub-branches each contain four members, which is similar to the results in other species. Predictive analysis of the SbEPF family gene promoter revealed the existence of multiple elements involved in plant response to stress, growth and development, hormone response, and photoperiod regulation, indicating the functional diversity of this gene family. SbEPFs have similar functions due to their conserved domains, but the diversity of non-conserved partial structures also gives them specificity [24,32].

Fluorescence quantitative analysis was utilized to investigate the tissue expression characteristics and response patterns to drought stress of *SbEPFs*. The *SbEPF9* and *SbEPF10* genes, which are homologous to the *AtEFP1* and *AtEPF2* genes that negatively regulate stomatal development in *Arabidopsis*, were induced to increase expression under moderate drought stress (Figure 7B). The *SbEPF3*, *SbEPF5*, *SbEPF8* and *SbEPF12*, which correspond to the CHAL subfamily in *Arabidopsis*, were highly expressed in roots and leaves (Figure 7A). It has been reported that this subfamily is mainly related to plant growth and flowering in *Arabidopsis* [33,34]. The results of the expression analysis showed that *SbCHALs* and *AtCHALs* exhibited different expression patterns in plant tissue, maybe due to the difference between monocotyledonous and dicotyledonous species. *SbEPF5* and *SbEPF12* had similar expression patterns in sorghum tissue and response to drought (Figure 7). Consistent with this, the two genes belonged to one sub-branch, indicating that they may have comparable functions (Figure 3). *AtEPFL9* is a positive stomatal development regulator of *Arabidopsis* [13,28]. *SbEPF6*, the *AtEPFL9* homologous gene, was significantly decreased in the drought response of the two varieties, suggesting that it may play a negative regulatory role in the drought response of sorghum. Other *SbEPF* family members showed varying degrees of response under drought treatment (Figure 7B). It was proved that *SbEPFs* were induced to express under drought stress and showed diverse expression levels, which indicated that SbEPFs may play different roles in plant growth and development. Although the above results indicated that SbEPFs are involved in the response of sorghum to drought stress, the underlying mechanism is still unclear. Previous studies have shown that EPFs have the function of regulating plant stomatal development [10,11,12,32]. Whether *SbEPFs* are involved in regulating sorghum stomatal development, how they participate, and the relevant regulatory networks require further research.

In the sorghum genome, we identified 12 members belonging to the EPF family and performed predictive analyses to explore their protein properties, chromosomal location, gene structure, evolutionary relationships, and promoter elements. These results indicated that SbEPF family members may be involved in plant growth and development as well as stress response. Our results provided important information on the functional role of *EPF* genes in sorghum plants. Integrating these analyses can aid in screening potential *SbEPF* candidate genes for further functional identification, and concurrently, aiming to improve the resistance of gramineous crops and ensure food security.

## 4. Materials and Methods

### 4.1. Genome-Wide Identification and Characterization of SbEPF Genes in Sorghum

The EPF Hidden Markov Model (HMM) profile of the EPF domain (pfam17181) was used to blast against the sorghum reference genome (Sorghum bicolor v3.1.1) to identify EPF genes in the sorghum genome. A total of 12 putative EPF-encoding genes were identified. In addition, the sequences were further confirmed conserved structures by using NCBI CD-Search (https://www.ncbi.nlm.nih.gov/Structure/cdd/wrpsb.cgi, accessed on 1 September 2022) and HMMER (https://www.ebi.ac.uk/Tools/hmmer/, accessed on 3 September 2022).The CDS lengths and the protein lengths, molecular weights, isoelectric points, and hydrophilicity analysis of the identified SbEPFs were conducted using ExPasy (https://web.expasy.org/protparam/, accessed on 10 October 2022).

### 4.2. Evolutionary Relationships of EPF Family Genes

Members of the family of *Arabidopsis* EPF sequence from TAIR (https://www.arabidopsis.org/index.jsp, accessed on 8 August 2022), and download the EPF sequences in different species, including *P. Trichocarpa*, *Zea Mays*, *Picea Abies*, *Oryza Sativa*, *Amborella*, from the PopGenie database (https://popgenie.org/?r=archive, accessed on 31 August 2022). All sequences have the corresponding conserved domains. The amino acids of all EPF target sequences were analyzed using the Neighbor joining (NJ) method in MEGA6 software to construct the phylogenetic tree.

### 4.3. Analysis of SbEPF Genes and Protein Structures

The gene structure map of SbEPFs and the conserved motifs and domains of candidate SbEPF proteins were identified and drawn by using TBtools (https://github.com/CJ-Chen/TBtools, accessed on 23 April 2023). The conserved motifs SbEPF proteins were analysed through WebLogo3 (http://weblogo.threeplusone.com/, accessed on 5 May 2023).

### 4.4. Analysis of cis-Element in SbEPF Promoters

The promoter sequences (2000 bp upstream of the start codon) of SbEPFs were analyzed online using Plant-CARE (http://bioinformatics.psb.ugent.be/webtools/plantcare/html/, accessed on 1 October 2022). All cis-regulatory elements were counted by Excel and subsequently visualized by Heatmapper (http://www.heatmapper.ca/, accessed on 28 May 2023).

### 4.5. Plant Materials and Drought Sample Collections

The drought-tolerant sorghum variety XN1 and the drought-sensitive variety Aihongmao were selected for their seed germination after which healthy and consistent seedlings were transplanted into pots (sand:vermiculite 1:1 *v*/*v*) and grown in a controlled environment [35]. Once the plants reached the three-leaf stage (approximately two weeks after planting), they were subjected to drought treatment. The most recently fully expanded leaves were collected at normal water supply (CK), moderate drought treatment (M), and heavy drought treatment (H). Different tissues of 2-week-old sorghum seedlings, including root, stem, and leaves, were collected. All the collected samples were immediately placed in liquid nitrogen for preservation at −80 °C.

### 4.6. RNA Extraction, Reverse Transcription, and qRT-PCR

Total RNA was extracted using an RNAprep Pure Plant Plus kit (Tiangen, Beijing), and reverse transcription was carried out according to the instructions of the FastKing RT kit. The reverse transcription product was mixed and stored on ice or at −20 °C. The cDNA was used as a template for qRT-PCR quantification. The RT-qPCR reaction mixture consisted of 10 μL SuperReal PreMix Plus, 1 μL cDNA template, and 0.6 μM of each primer, with RNase-free water added to a final volume of 20 μL, following the previous methods [26]. qRT-PCR amplification was performed using the SuperReal PreMix Plus according to the cycling parameters (95 °C for 15 min, 40 cycles of 95 °C for 10 s, and 60 °C for 30 s). *SbActin1* and *SbEIF4a* were used as the internal reference genes. Primer sequences were designed using DNAMAN5 software and are shown in Table S1.

### 4.7. Statistical Analyses

All data were analyzed using SPSS software (SPSS Inc., Chicago, IL, USA). Values were presented as the mean ± standard error (SE). Subsequent multiple comparisons were evaluated based on the One Way ANOVA test and two-tailed Student’s t-tests methods to calculate *p*-values [36].

## 5. Conclusions

In this study, we identified 12 SbEPF members in *sorghum.* These members were randomly distributed on eight chromosomes and were divided into four classes based on the phylogenetic tree. Gene structure and protein conserved motifs analysis demonstrated that SbEPF family members possess six to eight conserved cysteine residues at the C-terminus. The promoter *cis*-elements analysis of *SbEPFs* indicated that they may participate in plant growth and development, plant hormone response, and abiotic stresses. The qRT-PCR analysis showed that *SbEPFs* are expressed in different ways in sorghum tissues. Otherwise, the *SbEPF* genes may act as positive and negative regulators in response to drought stress in sorghum. The further research direction is to identify candidates for molecular breeding programs, conduct functional verification and mechanism exploration, and improve the crop drought resistance.

## Figures and Tables

**Table 1:** Physicochemical analysis of EPF family in Sorghum.

Name	Gene Model ID (V3.1.1)	CDS Length (bp)	Protein Length (aa)	Molecular Weight (KDa)	Protein Isoelectric Point (pI)	Chr. no.	Formula
SbEPF1	Sobic.001G106500	408	135	14.788	7.61	01	C_633_H_1025_N_197_O_198_S_7_
SbEPF2	Sobic.001G140400	369	122	12.905	9.81	01	C_555_H_906_N_178_O_161_S_8_
SbEPF3	Sobic.001G496400	393	130	13.932	9.24	01	C_622_H_964_N_176_O_169_S_10_
SbEPF4	Sobic.002G025300	480	159	17.445	9.04	02	C_747_H_1227_N_231_O_224_S_13_
SbEPF5	Sobic.003G339600	435	144	15.893	9.28	03	C_684_H_1069_N_213_O_188_S_19_
SbEPF6	Sobic.003G399800	372	123	13.486	8.78	03	C_586_H_938_N_170_O_171_S_12_
SbEPF7	Sobic.004G229700	335	111	11.784	9.24	04	C_504_H_813_N_153_O_153_S_10_
SbEPF8	Sobic.005G166500	441	146	15.689	11.51	05	C_672_H_1105_N_221_O_183_S_15_
SbEPF9	Sobic.006G104400	501	166	18.062	9.22	06	C_778H1239N243_O_226_S_14_
SbEPF10	Sobic.006G233600	618	205	21.524	9.07	06	C_916_H_1513_N_279_O_276_S_21_
SbEPF11	Sobic.007G197500	441	146	15.566	8.83	07	C_658_H_1076_N_208_O_207_S_11_
SbEPF12	Sobic.009G173200	510	169	18.181	9.64	09	C_799_H_1268_N_236_O_226_S_12_

## Data Availability

All data in this study are available in this article and its Supplementary Files.

## Supplementary Materials

The following supporting information can be downloaded at: https://www.mdpi.com/article/10.3390/plants12223912/s1, Supplementary Information S1: SbEPF protein sequences; Supplementary Information S2: SbEPF CDS sequences; Supplementary Information S3: Promoter analysis of SbEPFs; Table S1: List of all primers used in this study.
