# Supplementary material for: Genome-Wide Identification and Analysis of the EPF Gene Family in Sorghum bicolor (L.) Moench
Source: Plants (Basel). 2023 Nov 20;12(22):3912. doi: 10.3390/plants12223912 (PMC10674733; doi:10.3390/plants12223912)
Supplement: Supplementary file 1 [file plants-12-03912-s001.zip › Information S1-SbEPFs protein sequences.pdf]

S1. SbEPFs protein sequences.

Sobic.001G106500（SbEPF1）

MELQLYYSTSLLLLLLLLPSSSSSHGLRSADGTRALHYRLKDPHPPKVGEGAA
ESLIGSRPPRCDGKCAPCGRCEAVQVPVAPRVDSRAGEGDADEPRRRGRDGL
LLGSVDEESYTDYKPLNWRCRCADRRALDP*

Sobic.001G140400 （SbEPF2）

MGHIFLLLVVLLLTSTRATAAVHARASAIEEASFAGIRGVIGSRPPSCAGRCRS
CGHCEAVQVPVSPQQLQRKKKEGLGHSSRAAAAAATTGGRAMPASYDDHS
NYKPLSWRCKCGRHILDP*

Sobic.001G496400 （SbEPF3）

MGWPPSCGLGRRRRGLSATLPLFVVVLLLLLLFFSTPGTCGTPAKGSGGLVAP
PTTTTEEEDGYSWSWDPAAARRGLVGPGSSPPTCRSRCGGCHPCRPVHVAIQ
PGRSFPLEYYPEAWRCKCGNKLFMP*

Sobic.002G025300 （SbEPF4）

MVHLLQCRRCSGSVHKLFLFSILLLLTSSMAVAFSDASDTRRLPLMLQPEDKE
AVAADDKQPLEAGGGRRSRSGETTEARRRRGLIGSRPPRCERVCMSCGHCEA
VQVPIVPQHEEEKARASASAVTLAAAMFTYRVDGISNYKPLSWKCRCGGTIL
DP*

Sobic.003G339600 （SbEPF5）

MECSRGRRRWRWCGRSMLKLAGLCCFAVAIVICFCGVRSWACSASACRGRS
TVLLRSEPWGRGRAAASCDNQGCYINTGVQGQGQWRRLLAEGPGSYPPRCT
SKCGDCSPCYPVHVAVPPGVPVTTEYYPEAWRCKCGNRLYMP*

Sobic.003G399800 （SbEPF6）

MANGCPTTTTSSLLLFFLLSCLLIGHALCSQGHNGRTSGADSVVQYPHQELPA
KHIVLQEAVKGLNKGILSKYTRRMLIGSIAPICTYNECRGCRFKCTAEQVPVD
ANDPMNSAYHYKCVCHR*

Sobic.004G229700 （SbEPF7）

MAVSCSPRRALIAAVSLCFLLGAATSIRTATFSPSQNLAEDKSRLGSTPPSCHN
RCSACNPCMPVQVTTAPGLGRAARVADDTVTVAGFSRYSNYKPLGWKCRC
DGRLYDP*

Sobic.005G166500 （SbEPF8）

MMGAVTCRSRTSRRRCHAATALLAAVLFAAAMVVATGRPVRLPAAALARR
RIDSSSTAMIMNAGGRAATTTTRWTAGAAMDDVLPAAARRRWLVGPGSSPP
TCRARCGRCTPCRPTRVAIQPGVGPQWEYYPEVWRCKCGNKLFMP*

Sobic.006G104400 （SbEPF9）

MRMRESSACRCRPRIWWWSPAPAAAVVLVLLLLITTSSSTHVADGGVRTTPR
AYGPGDAGVLDDAEAPAAAGGARRASSRATYKFPADRTSEDEGSLVVVEED
RRWWLKDRAATGSRLPDCAHACGPCSPCRRVIVSFRCALMASESCPIAYRCM
CRGRFFRVPSP*

Sobic.006G233600 （SbEPF10）

MLAIAAAIYMCTLGAETTYVQVQLARPRLAQHWSNRALRCLPGSRSQCSAA
ATPRRRLSSQHCEAYAVSLGLMGRHAGVVLLALTVVLLLAAVGDGIRPAPTA
GASEMVHGSTTTTTEMVVVAAPSAAQVQGKRSRGGKDDDLVLREEVVRAT
GSSLPDCSHACGACSPCSRVMVSFKCSASEPLPCPMVYRCMCRGKCYPVPSS*

Sobic.007G197500 （SbEPF11）

MAAAYGSTRQQQQLRRCVALCCLLLVAVLVIIGYAVLADARAGSPRTDEVA
LQTGQLQGFSRRSSTEALTTTTTTAAAGGWTALDTEQQAAAGGGGRRRMLV
GSRAPTCTYNECRGCRHRCSVQEVPIDASDPINSAYHYKCICHL*

Sobic.009G173200 （SbEPF12）

MVVQVMPPPLLMVGRQQPRWWWWRGSSGSARSTTLGGVAVVVALLVSLC
FIGSRFTGTQLGGCSTLAALASSDSGRTTVAAAAGAAHPGGQVVATDETQEE
RVYYIIARRRRRLLSGGLGSHPPRCTTKCGSCNPCYPVHVSVPPGVLVTTEYY
PEAWRCKCRNQLYMP*
