# Supplementary material for: Genome-Wide Identification and Analysis of the EPF Gene Family in Sorghum bicolor (L.) Moench
Source: Plants (Basel). 2023 Nov 20;12(22):3912. doi: 10.3390/plants12223912 (PMC10674733; doi:10.3390/plants12223912)
Supplement: Supplementary file 1 [file plants-12-03912-s001.zip › Information S2-SbEPFs CDS sequences.pdf]

S2. SbEPFs CDS sequences.

Sobic.001G106500（SbEPF1）

ATGGAACTCCAGCTCTACTACTCCACATCCCTGCTGCTGCTCCTCCTCCTGC
TACCCTCTTCCTCTTCCTCCCACGGCCTGCGCTCTGCCGACGGTACTAGAGC
GCTGCATTACCGGCTCAAGGATCCGCATCCACCAAAGGTCGGCGAGGGGG
CGGCGGAGTCGCTGATCGGGTCAAGGCCGCCGCGGTGCGACGGCAAGTGC
GCGCCGTGCGGGCGGTGCGAGGCGGTGCAGGTGCCGGTGGCGCCGCGGG
TCGACAGCCGCGCGGGCGAGGGCGACGCCGACGAGCCCCGGCGCCGCGG
CCGCGACGGGCTGTTGTTGGGCAGCGTCGACGAGGAGAGCTACACCGACT
ACAAGCCTCTCAACTGGAGGTGCCGGTGCGCGGACCGGCGAGCCCTGGAT
CCGTGA

Sobic.001G140400 （SbEPF2）

ATGGGCCATATTTTCCTCCTCCTGGTAGTCCTCCTGCTCACTTCCACGCGAG
CCACCGCCGCCGTCCATGCCAGAGCTTCCGCCATTGAGGAGGCGAGCTTTG
CAGGCATCAGGGGCGTGATCGGATCCCGGCCGCCGAGCTGCGCGGGGAGG
TGCAGGTCCTGTGGCCACTGCGAGGCAGTGCAGGTGCCCGTATCTCCGCA
GCAGCTGCAGAGGAAGAAGAAGGAGGGTCTCGGCCATAGCAGCAGAGCT
GCTGCTGCTGCTGCTACCACCGGTGGAAGAGCAATGCCGGCCTCCTACGAC
GACCACTCCAACTATAAGCCGCTGAGCTGGAGATGCAAATGTGGGCGGCA
CATCCTCGACCCTTGA

Sobic.001G496400 （SbEPF3）

ATGGGCTGGCCGCCGAGCTGCGGCTTGGGCCGGCGACGACGGGGCCTATC
GGCCACGCTGCCGCTCTTCGTCGTCGTCCTCCTCCTCCTGCTGCTCTTCTTC
TCCACCCCCGGGACGTGTGGCACGCCGGCGAAGGGGAGCGGCGGCTTGGT
GGCGCCGCCGACGACGACGACGGAGGAGGAGGACGGGTACTCCTGGTCC
TGGGACCCGGCGGCGGCGCGCCGGGGGCTGGTGGGGCCGGGGTCGTCGC
CGCCCACCTGCCGCAGCCGCTGCGGGGGCTGCCACCCCTGCCGACCCGTC
CACGTCGCCATCCAGCCAGGCCGGAGCTTCCCGCTCGAGTACTACCCGGA
GGCCTGGCGCTGCAAGTGCGGCAACAAGCTCTTCATGCCCTGA

Sobic.002G025300 （SbEPF4）

ATGGTCCATCTGTTGCAATGCAGACGATGCAGCGGCAGTGTTCACAAACTC
TTCCTCTTCTCTATCCTCCTCCTCCTCACATCTTCCATGGCGGTAGCATTCTC
AGATGCCTCAGATACCAGGAGGTTGCCGCTGATGCTACAGCCGGAAGACA
AGGAGGCAGTGGCCGCTGATGACAAGCAGCCGCTGGAGGCGGGAGGAGG
AAGAAGAAGCAGATCAGGGGAGACTACGGAAGCAAGGCGGCGGCGTGGT
CTGATCGGATCGAGGCCCCCGAGATGCGAGCGGGTGTGCATGTCCTGCGGC
CACTGCGAGGCGGTGCAGGTGCCCATCGTGCCACAGCATGAGGAGGAGAA
GGCGAGAGCCTCGGCGTCGGCGGTCACCCTCGCCGCCGCCATGTTCACCTA
CCGGGTGGACGGCATCAGCAACTACAAGCCGCTGAGCTGGAAGTGCAGGT
GTGGAGGAACCATCCTGGATCCATGA

Sobic.003G339600 （SbEPF5）

ATGGAGTGCTCCCGGGGGAGGCGGAGATGGAGGTGGTGTGGCAGATCCAT
GCTAAAGCTGGCGGGGCTATGCTGCTTCGCCGTGGCCATCGTCATCTGCTT
CTGCGGCGTTCGGTCCTGGGCTTGCAGTGCCAGCGCCTGCAGGGGGAGGA
GTACTGTGTTGCTCCGTTCAGAACCATGGGGACGAGGACGAGCGGCAGCA
TCTTGCGATAACCAGGGGTGCTACATCAACACGGGCGTGCAAGGGCAAGG
GCAATGGCGGCGGCTGCTGGCGGAAGGTCCGGGGTCATACCCGCCACGGT
GCACGTCCAAGTGCGGCGACTGCAGCCCGTGCTACCCGGTGCACGTGGCC
GTGCCGCCGGGCGTGCCGGTCACCACCGAGTACTACCCGGAGGCGTGGAG
GTGCAAGTGCGGCAACCGCCTCTACATGCCATGA

Sobic.003G399800 （SbEPF6）

ATGGCTAATGGTTGCCCCACAACTACCACAAGCTCCCTGCTCCTCTTCTTCC
TTCTCTCTTGCCTGCTCATCGGCCATGCTCTCTGCAGCCAAGGCCACAATG
GCAGAACATCAGGTGCTGATTCTGTGGTGCAATATCCCCACCAGGAATTGC
CTGCTAAACATATAGTTTTGCAGGAGGCCGTCAAGGGTCTAAACAAGGGCA
TACTATCGAAGTATACAAGGAGAATGTTGATCGGCTCAATTGCTCCAATATG
CACATACAACGAGTGCAGGGGGTGTCGATTCAAGTGTACTGCTGAGCAAG
TCCCAGTGGATGCAAATGACCCCATGAACAGTGCTTACCACTACAAGTGTG
TTTGCCACAGGTGA

Sobic.004G229700 （SbEPF7）

ATGGCCGTGAGCTGCTCACCTCGCCGAGCCCTCATCGCCGCCGTGTCCCTC
TGCTTCCTTCTCGGCGCCGCGACCAGCATCCGCACCGCCACGTTTTCCCCT
TCTCAGAACCTGGCGGAGGACAAGTCGCGGCTGGGGTCGACGCCGCCGA
GCTGCCACAACCGGTGCAGCGCCTGCAACCCCTGCATGCCCGTCCAGGTG
ACGACCGCGCCGGGGCTCGGCCGCGCGGCGCGCGTCGCCGACGACACGG
TGACGGTTGCCGGCTTCTCGCGCTACTCCAACTACAAGCCGCTGGGGTGGA
AATGCCGCTGCGACGGCCGCCTGTACGACCCCTAG

Sobic.005G166500 （SbEPF8）

ATGATGGGCGCCGTGACCTGCCGGAGCCGGACGAGCCGGCGCCGCTGCCA
CGCCGCCACCGCCCTCCTCGCCGCCGTCCTCTTCGCCGCGGCCATGGTTGT
GGCAACAGGGCGGCCGGTCCGTTTGCCAGCGGCGGCGCTGGCGAGGAGG
CGGATAGACTCGTCGTCGACGGCGATGATCATGAACGCCGGCGGCAGGGC
AGCGACCACCACCACGAGGTGGACAGCAGGCGCCGCCATGGACGACGTC
CTCCCAGCAGCAGCCCGGCGGCGGTGGCTGGTGGGTCCGGGGTCGTCGCC
GCCGACGTGCCGCGCGCGGTGCGGGCGGTGCACGCCGTGCCGGCCCACCC
GCGTGGCCATCCAGCCCGGCGTCGGACCCCAGTGGGAGTACTACCCGGAG
GTGTGGCGCTGCAAGTGCGGCAACAAGCTCTTCATGCCATGA

Sobic.006G104400 （SbEPF9）

ATGAGAATGCGGGAGTCCTCTGCTTGCCGGTGCCGGCCGAGGATATGGTGG

TGGTCTCCAGCCCCAGCAGCAGCGGTCGTGTTGGTGCTCCTGCTGCTCATC
ACCACTTCGTCGTCAACGCACGTTGCCGACGGCGGCGTCAGGACGACACC
ACGTGCGTACGGTCCAGGTGACGCCGGCGTGCTCGACGACGCGGAGGCAC
CGGCTGCTGCTGGTGGTGCTCGTCGCGCAAGCAGCAGGGCAACCTACAAA
TTTCCAGCAGACCGGACTAGCGAGGACGAAGGCTCGCTGGTGGTGGTGGA
GGAGGATCGGCGGTGGTGGCTGAAGGATCGCGCGGCGACGGGGTCGCGG
CTGCCGGACTGCGCGCACGCGTGCGGGCCGTGCTCCCCGTGCCGGCGCGT
CATCGTGAGCTTCAGGTGCGCGCTGATGGCCTCCGAGTCCTGCCCCATCGC
CTACCGCTGCATGTGCCGCGGCAGGTTCTTTCGGGTGCCCTCCCCCTAG

Sobic.006G233600 （SbEPF10）

ATGCTGGCTATAGCTGCGGCTATATATATGTGTACGCTCGGCGCGGAGACGA
CGTACGTGCAGGTGCAGCTAGCCCGGCCTCGCCTCGCGCAACATTGGAGC
AACCGCGCGCTTCGTTGCCTCCCGGGCTCTCGCTCTCAGTGCTCCGCCGCC
GCCACGCCGCGACGACGTCTCAGCAGCCAGCACTGCGAAGCGTACGCGGT
CTCTCTTGGGCTGATGGGGAGGCACGCTGGCGTCGTCCTTCTTGCTCTGAC
GGTAGTGCTGCTGCTCGCCGCCGTGGGTGATGGCATCAGACCAGCTCCTAC
TGCTGGGGCGAGCGAGATGGTGCATGGATCGACGACGACGACGACGGAGA
TGGTCGTCGTAGCTGCGCCGTCAGCTGCACAGGTGCAGGGGAAGAGGAGC
AGGGGCGGCAAGGACGACGACCTGGTGCTGCGGGAGGAGGTGGTGCGCG
CGACGGGGTCGAGCCTCCCGGACTGCTCGCACGCGTGCGGGGCGTGCTCG
CCGTGCAGCCGCGTCATGGTCAGCTTCAAGTGCTCCGCGTCCGAGCCGCTG
CCGTGCCCCATGGTGTACCGCTGCATGTGCAGGGGCAAGTGCTACCCGGTG
CCATCCAGCTGA

Sobic.007G197500 （SbEPF11）

ATGGCAGCAGCATATGGAAGCACCAGGCAGCAGCAGCAGTTGCGTCGTTG
TGTGGCGCTGTGCTGTCTCCTCCTCGTGGCTGTCCTAGTGATTATTGGTTAT
GCCGTGTTAGCTGATGCTCGAGCAGGCTCGCCGCGGACTGACGAAGTAGC
ACTTCAGACAGGGCAGCTGCAGGGATTTAGTAGGAGATCGAGTACTGAAG
CACTGACGACGACGACGACGACGGCTGCAGCTGGTGGTTGGACGGCGTTG
GACACGGAGCAGCAGGCGGCTGCCGGCGGCGGCGGGAGGAGGAGGATGC
TGGTCGGGTCCAGGGCTCCGACGTGCACCTACAACGAGTGCCGAGGCTGC
CGGCACAGGTGCAGCGTGCAGGAGGTGCCCATCGACGCCAGCGACCCCAT
CAACAGCGCCTACCACTACAAATGCATCTGCCATCTATAG

Sobic.009G173200 （SbEPF12）

ATGGTGGTTCAGGTGATGCCGCCGCCGCTGCTCATGGTGGGGAGGCAGCA
GCCGAGGTGGTGGTGGTGGAGGGGGAGCTCCGGATCCGCGCGGTCGACG
ACGCTCGGCGGCGTCGCGGTGGTGGTTGCGCTGCTCGTCTCCCTCTGCTTC
ATCGGCAGCCGTTTCACGGGGACACAGCTGGGTGGCTGCAGTACCCTCGC
AGCACTCGCTTCATCAGATTCCGGGAGGACGACCGTGGCAGCGGCAGCAG
GAGCAGCCCATCCCGGCGGTCAGGTGGTGGCGACTGATGAGACGCAGGAG
GAGCGGGTTTATTACATCATCGCGCGACGACGACGCCGGCTGCTGTCGGGC

GGGCTGGGGTCGCATCCGCCACGGTGCACCACCAAATGCGGCAGCTGCAA
CCCGTGCTACCCGGTGCACGTGTCCGTGCCGCCGGGGGTTCTGGTCACCAC
CGAGTACTACCCGGAGGCGTGGCGGTGCAAGTGCCGGAACCAGCTCTACA
TGCCATGA
