# Supplementary material for: Genome-Wide Identification and Analysis of the EPF Gene Family in Sorghum bicolor (L.) Moench
Source: Plants (Basel). 2023 Nov 20;12(22):3912. doi: 10.3390/plants12223912 (PMC10674733; doi:10.3390/plants12223912)
Supplement: Supplementary file 1 [file plants-12-03912-s001.zip › Table S1. List of all primers used in this study.pdf]

Table S1. List of all primers used in this study.

Name	Sequence (from 5' to 3')
β-SbActin1-qF	ATCGAGCACGGAATCGTCAG
β-SbActin1-qR	GACCGCTGGCATACAGAGAG
SbEIF4a-qF	AGGATTGGCACCAGAAGGGT
SbEIF4a-qR	CACATCAAGCCCCTTGCAGA
SbEPF1-qF	GGAGTCGCTGATCGGGTCAA
SbEPF1-qR	GTTGAGAGGCTTGTAGTCGGT
SbEPF2-qF	AGAAGAAGGAGGGTCTCGGC
SbEPF2-qR	TCAAGGGTCGAGGATGTGCC
SbEPF3-qF	GACGGGTACTCCTGGTCCT
SbEPF3-qR	GTAGTACTCGAGCGGGAAGC
SbEPF4-qF	AGGGGAGACTACGGAAGCAA
SbEPF4-qR	CGGCTTGTAGTTGCTGATGC
SbEPF5-qF	CCAGGGGTGCTACATCAACA
SbEPF5-qR	ATGGCATGTAGAGGCGGTTG
SbEPF6-qF	GGCCGTCAAGGGTCTAAACA
SbEPF6-qR	TCACCTGTGGCAAACACACT
SbEPF7-qF	CAGAACCTGGCGGAGGACAA
SbEPF7-qR	CGGCTTGTAGTTGGAGTAGCG
SbEPF8-qF	CACGAGGTGGACAGCAGG
SbEPF8-qR	CATGAAGAGCTTGTTGCCGC
SbEPF9-qF	GGTGGTGGCTGAAGGATCG
SbEPF9-qR	GAGGGCACCCGAAAGAACCT
SbEPF10-qF	TGCTGGCTATAGCTGCGG
SbEPF10-qR	GGAGCACTGAGAGCGAGAG
SbEPF11-qF	GCGGACTGACGAAGTAGCAC
SbEPF11-qR	TCGGCACTCGTTGTAGGTG
SbEPF12-qF	GCAGGAGGAGCGGGTTTATT
SbEPF12-qR	TGGCATGTAGAGCTGGTTCC
